# Co-created Technological Solutions for Caregivers in Health Care: Systematic Review

**DOI:** 10.2196/41260

**Published:** 2023-05-01

**Authors:** Jose Antonio Merchán-Baeza, Cristina Borralleras Andreu, Eduard Minobes-Molina, Sergi Grau Carrión, Montse Romero-Mas, Anna Ramon-Aribau

**Affiliations:** 1 Methodology, Methods, Models and Outcomes of Health and Social Sciences Faculty of Health Sciences and Welfare, Centre for Health and Social Care Research University of Vic-Central University of Catalonia Vic Spain; 2 Digital Care Research Group Faculty of Science, Technology and Engineering, Centre for Health and Social Care Research University of Vic-Central University of Catalonia Vic Spain

**Keywords:** co-creation, technological solutions, caregivers, health care, systematic review, mobile applications

## Abstract

**Background:**

Support interventions for caregivers can reduce their stress, possibly improving the quality of patients’ care while reducing care costs. Technological solutions have been designed to cover their needs, but there are some challenges in making them truly functional for end users. Co-design approaches present important opportunities for engaging diverse populations to help ensure that technological solutions are inclusive and accessible.

**Objective:**

This study aimed to identify co-created technological solutions, as well as the process followed for their co-creation, in the field of health for caregivers.

**Methods:**

The literature review was conducted in the Medline, Web of Science, Scopus, Science Direct, Scielo, and IEEE Xplore databases. The inclusion criteria were studies written in English or Spanish and with a publication date until May 2021. The content had to specify that the caregivers actively participated in the co-creation process, which covered until the development phase of the technological solution (prototype). The level of evidence and the methodological quality were analyzed when possible, using the Scottish Intercollegiate Guidelines Network criteria and the Mixed Methods Appraisal Tool, version 2018, respectively.

**Results:**

In total, 410 papers were identified, and 11 met the eligibility criteria. The most predominant articles were mixed methods studies and qualitative studies. The technology used in the analyzed articles were mobile or web applications (9 studies) and specific devices such as sensors, cameras, or alarm systems (2 studies) to support the health and social aspects of caregivers and improve their education in care. The most common patient profile was older people (7 studies); 6 studies used co-creation in the requirements phase, 6 studies detailed the design phase. In 9 studies, the prototype was iteratively refined in the development phase, and the validation phase was performed in 5 of the reviewed studies.

**Conclusions:**

This systematic review suggests that existing co-created technological solutions in the field of health for caregivers are mostly mobile or web applications to support caregivers’ social health and well-being and improve their health knowledge when delivering care to patients, especially older people. As for the co-creation process, caregivers are particularly involved during development and in the design. The scarce literature found indicates that further research with higher methodological quality is needed.

## Introduction

The steady increase in the number of people with acute and chronic diseases and increasing life expectancy place new demands on the health systems [1]. This increase is linked to a higher demand for care, representing a significant overload on public health resources [2]. At the same time, the demand for informal care is likely to increase over the coming decades [3]. In fact, caregivers have a ubiquitous and very substantial presence throughout the world, including the following countries: 43.5 million caregivers in the United States, 8.1 million caregivers in Canada, 6.5 million caregivers in the United Kingdom, and more than 8 million caregivers in France [4]. It is estimated that 10%-25% of Europe’s population regularly provides informal care, even though one’s identification as a caregiver and the definitions of caregivers vary in different contexts [3].

Informal caregivers usually are family members, neighbors, close acquaintances, or other significant individuals who provide unpaid daily assistance to a family member or dependent older adult who cannot care for themselves [5]. They play a strategic role in the daily activities of their dependent care recipients; however, informal care negatively affects the caregiver’s work productivity and their health, leading to a gradual worsening of caregivers’ quality of life. A survey conducted in the United States demonstrated that 32% have high caregiver burden and 19% have medium caregiver burden based on a measurement of time spent providing care and the care recipient’s degree of dependency [6]. Considering this, it is clear that many caregivers need support services to improve their health and quality of life [7].

Lack of support is a significant problem [8], and caregivers’ demand for education related to functional care is high [9]. A current systematic review concluded that support interventions for caregivers can reduce their stress, possibly improving the quality of patients’ care while reducing care costs [10]. Consequently, it is essential to introduce user-friendly and time-effective educational and supportive interventions.

Technological health solutions, especially in the form of assistive technologies, create significant opportunities to optimize both health and social care delivery. In this paper, we consider technological health solutions for caregivers as those that can transform and complement current care such as web or mobile applications, artificial intelligence, or virtual or augmented reality that can be used for medication management, community support, cognitive stimulation, or emotional support; nevertheless, we do not consider static repositories of information (such as static web pages or blogs) as a technological solution. Many studies support the idea that technological solutions can support conventional health care provision methods, thereby reducing demand for local services [11]. Today, technological solutions are popular [12], and they have the potential to provide personalized health care and disease management strategies and services to patients and their family members, as well as offer a flexible mode of communication between health workers and their consumers [13,14].

Lately, there has been a shift in the development of new products, first from a supplier-centered design (ie, service providers design a product) to user-centered design (ie, based on the user’s needs) and now to co-design, also called co-creation. In co-creation, designers, service providers or suppliers, and consumers work together to identify the problems and design solutions [15]. To achieve better outcomes, all parties have an active role [16], contributing and working together by using their knowledge and resources [17]. Co-creation in health interventions involves the equal partnership of the people who engage in a health intervention, such as service suppliers (ie, health staff), end users (ie, patients, families, and caregivers), and intervention developers (eg, information technology experts) [15]. Recently, this method has been widely used to develop health interventions [15], more in the preparation of recommendations [18], evaluation frameworks [19], or the creation of new knowledge [20] and not so much in the technological field.

The concerns of caregivers have been reported from many points of view, from sociological issues, national regulation, and stakeholders’ views, to caring activities to avoid the negative effect of losing control due to informal care and the so-called caregiver burden [21,22]. Technological solutions have been designed consistent with the current context, which cover some of these needs and present some challenges to make them truly functional for end users and caregivers. Co-creation might be a good option [23], but there is still a gap in identifying the type of technological solutions developed and their characteristics and in measuring their co-creation development.

This systematic review aimed to identify co-created technological solutions, as well as the process followed for their co-creation, in the field of health for caregivers.

## Methods

### Study Design

This systematic review was carried out following the PRISMA (Preferred Reporting Items for Systematic Reviews and Meta-Analysis) guideline [24]. The research questions from which this systematic review started were the following: What co-created technological solutions exist for caregivers in the field of health? What co-creation process is followed for the design and development of this technology?

### Search Strategy

The literature review was conducted in the Medline, Web of Science, Scopus, Science Direct, Scielo, and IEEE Xplore databases. The main search terms used to carry out this work were co-creation AND technology AND health AND caregiver. The full search string is available in Multimedia Appendix 1.

### Eligibility Criteria

The inclusion criteria were studies written in English or Spanish and with publication dates until May 2021. No starting year was established for the search in order to cover all existing evidence published by journals over all time. The included studies had to specify that the caregivers actively participated in the co-creation process, which covered until the development phase of the technological solution (prototype). The publications that were exclusively aimed at the people being cared for were excluded; only publications aimed at the population of caregivers were included. All publications that did not appear in peer-reviewed journals were excluded, except those extracted from the IEEE Xplore database, due to the relevance and impact of contributions to conferences in the field of technological engineering.

### Study Selection and Data Extraction

Initially, the search was carried out in the different databases by 2 researchers from the group (CBA and MRM). Subsequently, an independent blinded review process in which the different researchers of the team participated was carried out. The screening phase began with an independent blinded review of the previously identified studies by 2 other investigators. First, 2 researchers (SGC and EMM) evaluated the titles and the abstracts of the studies to assess their eligibility. Second, the remaining article’s full texts were assessed by the other 2 researchers (ARA and JAMB). In the 3 phases, the disagreements in selecting the studies between the 2 researchers were resolved in consensus by consulting the full text again. When the disagreements persisted, a third reviewer of the team assessed the eligibility of the research. Finally, in those studies in which the design could be evaluated, the level of evidence and methodological quality were independently analyzed using the Scottish Intercollegiate Guidelines Network (SIGN) criteria [25] and the Mixed Methods Appraisal Tool [26], version 2018, respectively. In both cases, disagreements were also resolved by consensus.

Independently and in pairs, using a template, the researchers extracted the following descriptive information from the articles included in the systematic review: authors and location, design, level of evidence, methodological quality, patient profile, type of technology designed, and objective of the designed technology. In addition, detailed information was extracted related to the co-creation process (name of the phase, description of the phase, agents involved, and result variables), divided into 4 phases: (1) requirements, (2) design, (3) development, and (4) validation. These phases were extracted from the studies included in the systematic review as common points between the different frameworks used in them [27]. The requirements phase consisted of identifying the problem and setting the objectives of the process. The design phase consisted of the creation of a solution. The development phase consisted of the implementation of a functional prototype. Finally, the validation phase consisted of evaluating the co-creation process and the effectiveness of the proposed solution.

Based on these categories to extract the information, 2 tables of results were created to subsequently analyze the content with the aim of answering the research questions initially raised and, therefore, the objectives of this systematic review.

## Results

### Main Results

**Figure 1:**
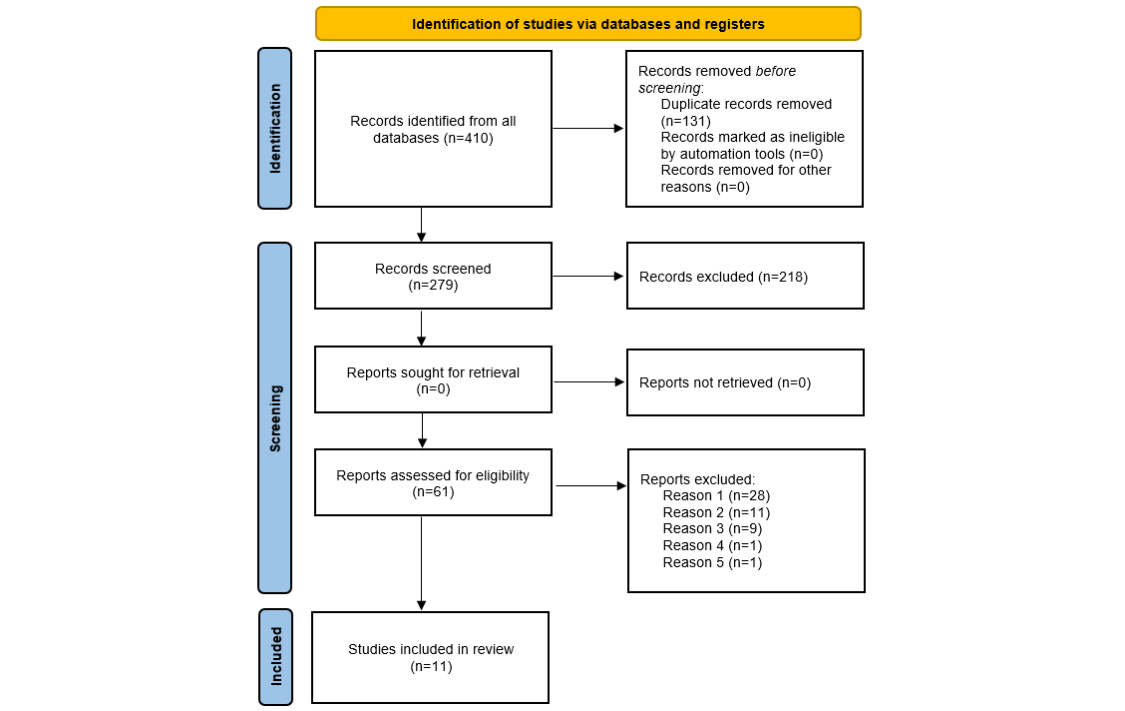
PRISMA (Preferred Reporting Items for Systematic Reviews and Meta-Analyses) flowchart displaying the different stages of the screening process. Reason 1: not developing the technology; Reason 2: not including caregivers as their target group; Reason 3: not having a participatory design; Reason 4: part of another study already included; Reason 5: not having enough information to determine compliance with the inclusion criteria.

The search produced 410 papers. We removed duplicates, leaving 279 papers. Titles and abstracts were screened to ensure alignment with the inclusion criteria, and 218 were eliminated from the study, thus leaving 61 eligible papers for further scrutiny. We read the entire text of 61 papers to assess eligibility in line with the inclusion and exclusion criteria. We eliminated 50 of the publications mainly because the studies were not consistent with our inclusion criteria; the reasons included not developing the technology (reason 1), not including caregivers as their target group (reason 2), not having a participatory design (reason 3), being part of another study already included (reason 4), or not having enough information to determine compliance with the inclusion criteria (reason 5). Figure 1 indicates the process of searching and identifying the papers through the PRISMA flow diagram.

### Descriptive Information

Authors and location, design, level of evidence, patient profile, type of technology designed, and objective of the designed technology are detailed in Table 1.

**Table 1:** Descriptive data.

Author(s), year (location)	Design	Level of evidence^a^	Patient profile (age)	Type of technology designed	Purpose of the designed technology
Backman et al, 2020 (Canada) [28]	Mixed methods study	—^b^	Older adults leaving hospital (67-97 years)	Mobile/web application	To manage the personalized needs of geriatric rehabilitation patients during their transition from the hospital to home
Egan et al, 2021 (United Kingdom) [29]	Mixed methods study	—	Caregivers (NR^c^)	Mobile/web application	To educate and support caregivers in the undertaking of regular physical activity at home during and beyond COVID-19 restrictions
de la Harpe and van Zyl, 2011 (South Africa) [30]	Mixed methods study	—	Population from under-resourced communities (NR)	Mobile/web application	To participate as simultaneous producers and consumers of information, relating to their own experiences, and to contribute to a joint repository of information and educational material in their own "idiom"
Guerrero et al, 2019 (Sweden) [31]	Case study	3	Older adults (57-67 years)	Specific devices	To assist with medication management
Harding et al, 2021 (India, Uganda, Zimbabwe) [32]	Mixed methods study	2	Palliative patients (NR)	Mobile/web application	To improve access to palliative care
Latulippe et al, 2020 (Canada) [33]	Qualitative study	—	Functionally dependent older persons (NR)	Mobile/web application	To facilitate the process of help-seeking for caregivers of functionally dependent older persons
Lemetyinen et al, 2018 (United Kingdom) [34]	Randomized controlled trial	1+	African Caribbean persons diagnosed with schizophrenia or other nonaffective psychosis (NR)	Mobile/web application	To improve knowledge about and attitudes toward schizophrenia in African Caribbean families
Meiland et al, 2014 (Netherlands and Germany) [35]	Qualitative study	—	People with mild cognitive impairment and dementia (NR)	Specific devices	To support community-dwelling people with mild cognitive impairment and dementia in daily functioning, monitor (deviations from) patterns in daily behavior, and automatically detect emergency situations
O'Connor, 2020 (United Kingdom) [36]	Qualitative study	—	People with dementia (NR)	Mobile/web application	To stimulate memory and communication by sharing memories together
Rathnayake et al, 2020 (Australia) [37]	Mixed methods study	—	People with dementia (NR)	Mobile/web application	To address functional disability care needs
Sin et al, 2019 (United Kingdom) [38]	Qualitative study	—	People with psychosis (NR)	Mobile/web application	To provide carers with psychoeducation and emotional support using health care professional contribution and peer support

^a^Scottish Intercollegiate Guidelines Network (SIGN) criteria: 1+ well-conducted meta-analyses, systematic reviews, or randomized controlled trials (RCTs) with a low risk of bias; 1- meta-analyses, systematic reviews, or RCTs with a high risk of bias; 2- case control or cohort studies with a high risk of confounding or bias and a significant risk that the relationship is not causal; 3 nonanalytic studies (eg, case reports, case series).

^b^Not able to be assessed.

^c^NR: not reported.

### Design, Level of Evidence, and Methodological Quality

Regarding the designs of the included articles, 5 mixed methods studies [28-30,32,37] and 4 qualitative studies [33,35,36,38] were the most predominant, followed by 1 randomized controlled trial (RCT) [34] and 1 case study [31]. The level of evidence in only 3 of the 11 articles could be assessed using the SIGN criteria [25], with the following results: 1 level 1+ for the RCT study [34], 1 level 2- for a mixed methods study [32], and 1 level 3 for a case study [31]. The methodological quality assessment indicates that only 3 studies fully met the quality criteria. The results are available in Multimedia Appendix 2.

### Type of Technology

The technology used in the analyzed articles can be classified into mobile or web applications [28-30,32-34,36-38] and specific devices such as sensors, cameras, or alarm systems [31,35]. Mobile applications are applications that live and run on the device itself. Web applications are accessible through websites using a web browser and have functionality and interactive elements.

### Purpose of the Technological Solutions

The aims can be classified into education and information in health [28,29,34,37,38], social [28,37,38], and well-being [30,37,38] aspects; assistive systems [28,31,32,35]; and cognitive rehabilitation [36].

The main strategy for education and information is through content and resources (text, videos, storyboards) from the company’s or external sources’ solutions. Some of them use e-learning environments to introduce interactive resources such as quizzes [23,32]. They also use social interactions with experts or caregivers through forums, blogs, or peer support. The assistive systems aim to monitor and automatically detect emergencies or to improve patients’ autonomy. These systems use mobile or web applications in combination with specific devices. The data reported by the systems are shared with patients, family or community caregivers, or the patient’s care provider. Finally, 1 tool [36] provides a digital profile of the person with dementia to stimulate the person’s memory and improve communication with caregivers.

### Patient Profile

Most patient profiles in the articles were that of an elderly population, either being directly targeted [28,31,33] or including patients with health conditions that mostly affect older adults like dementia [35-37] or require palliative care [32]. The rest of the articles included patients with mental health conditions such as psychosis or schizophrenia [34,38] or other circumstances such as being from under-resourced communities [30].

### Co-creation

#### Co-creation — Agents Involved

The co-creation process involved a wide variety of agents. Caregivers, as a target group, were involved in all the studies (see Table 2). Other individuals involved in most studies were health professionals, who were included in 9 studies [28,29,31-33,35-38]; the research team (health, social, education, and technology researchers), who appeared in 8 studies [28-31,33,35,37,38]; patients, who participated in 6 studies [28,31,34-36,38]; and technology professionals, who participated in 6 studies [28-30,36-38]. Other agents who were involved in the co-creation process were community members in 3 studies [30,33,34] and students [30], social professionals [29], a volunteer [35], a museum manager [36], and a translator [30] in 1 study each. In addition, some heterogeneous groups also participated, such as an advisory committee [32,33] made up of community workers, health and social professionals, and caregivers; a young, mixed group [34] of relatives and patients; and a focus group [34] of relatives, caregivers, and patients. On average, between 4 and 5 different types of actors were consulted for each study.

**Table 2:** Involvement of informal caregivers in the co-creation phases.

Author(s), year (location)	Phase 1: requirements	Phase 2: design	Phase 3: development	Phase 4: validation
Backman et al, 2020 (Canada) [28]	—^a^	X^b^	X	X
Egan et al, 2021(United Kingdom) [29]	—	X	X	X
de la Harpe and van Zyl, 2011 (South Africa) [30]	—	X	—	—
Guerrero et al, 2019 (Sweden) [31]	X	—	—	X
Harding et al, 2021 (India, Uganda, Zimbabwe) [32]	—	X	X	X
Latulippe et al, 2020 (Canada) [33]	X	X	X	X
Lemetyinen et al, 2018 (United Kingdom) [34]	X	—	—	—
Meiland et al, 2014 (Netherlands and Germany) [35]	X	—	X	—
O'Connor, 2020 (United Kingdom) [36]	—	—	X	X
Rathnayake et al, 2020 (Australia) [37]	X	—	X	—
Sin et al, 2019 (United Kingdom) [38]	—	—	—	X

^a^Not included.

^b^Included.

With co-creation, users have an active role from the beginning of the creation process [39]. There are different frameworks for co-creation, but they have common phases [27]: requirements, design, development, and validation. The analysis of the process of creating the technological solution carried out in this systematic review is based and organized on these phases (Multimedia Appendix 3).

#### Co-creation — Phase 1: Requirements

The first phase of the co-creation process involves the requirements. Of the articles, 5 did not use co-creation in this phase [28,29,32,36,38]. The rest of the papers tackled this phase with a needs assessment [30,31,33-35,37]; 1 of these studies added a data gathering phase after the needs assessment [34]. Of the 6 articles that used a needs assessment, 5 specified the concrete methods used for the co-creation, while 1 only indicated that it was an ethnographic study [30]. Of the articles, 3 used interviews [31,35,37]. However, only 1 of these articles proceeded exclusively with interviews [31], while 1 study combined interviews with workshops, expert meetings, and consultation with partners [35] and the other study combined interviews with an online survey [37]. In addition, 1 more article adopted workshops [33], and another study used focus groups [34]. Finally, the article that added a data gathering phase introduced questionnaires for this purpose [34].

When we analyzed the participation of the agents by phase, 5 of the 11 selected articles incorporated caregivers [31,33-35,37], followed by health professionals [31,33,35], patients [31,34,35], and community members [30,33,34]. The research team was consulted in 2 of the 11 studies [30,33]. Only 1 of the articles included technology professionals [37] and students [30], and 5 articles did not mention involving any agent at this stage [28,29,32,36,38].

#### Co-creation — Phase 2: Design

The design phase of the co-creation process, the second of the 4 phases, was described in 6 of the 11 studies included in this systematic review. All stood out for the application of technology design methods, such as a modern agile and iterative co-design [28], the MoSCoW methodology [29], or the “keep, lose, change” approach [29], and specific design tools, such as service design methods [30], focusing on users’ needs during development. For this, different teamwork techniques were carried out, such as interviews [31,35], analysis [31], brainstorming [33], workshops [33,35], and debriefing [33], to reach agreement by consensus on aspects related to the technological solution such as the content, functionality, or appearance that would allow covering the previously identified needs.

When we analyzed the agents involved in this phase, the studies most frequently involved caregivers (6 studies) [28-30,32,33,35] and the research team [29-31,33,35] (5 studies), followed by health professionals in 4 studies [28,29,33,35]; patients in 2 studies [28,35]; and community members [33], technology professionals [29], and social professionals [29] in only 1 study each. In addition, 4 articles did not mention involving any agent at this stage [34,36-38].

#### Co-creation — Phase 3: Development

The development phase consists of the creation of a functional prototype. Unlike the proof of concept in the design phase, creation of the functional prototype is not only to answer technical and design questions. It must be functional and usable to test the fundamental hypotheses of the proposal. It consists of gradual and iterative development, alternating phases of creation, and testing for subsequent refinements. End users and co-creation participants generally participate in the testing phases, and the prototype is refined based on their feedback. In some cases, they may also participate in content development, design, and feature selection phases. They also usually participate by conducting a usability test, and the final prototype is refined based on their feedback.

In 1 study [34], the prototype was developed according to the results from the needs assessment and design phases, and the co-creation participants did not intervene; in another study [30], there was no description of the development. In 9 of the 11 studies, the prototype was iteratively refined, and co-creation participants were involved in all iterations [28,29,31-33,35,-38]. In 4 of these studies, caregivers’ and patients’ participation was limited to testing and feedback sessions, whereas in 4 of the studies, they were also involved in workshops on creating and selecting content and functionality [29,33,36,38]. In 1 study, a nurse was involved in gradual and iterative development [31]. Usability testing to refine the final prototype was conducted in 4 studies [28,29,35,38]. The number of iterations of the prototype development in the studies varied from 2 to 4 and was not detailed in 2 studies.

Analyzing agents involved in the 11 articles, caregivers were the most predominantly consulted, in 8 articles [28,29,32,33,35-38], followed by the research team in 7 articles [28-31,33,37,38]; health professionals in 7 articles [28,29,31,33,36-38]; technology professionals in 6 articles [28-30,36-38]; patients in 4 articles [28,35,36,38]; and community members [33], social professionals [29], students [30], volunteers [35], and translators [30] in at least 1 article each. In addition, 1 article did not mention involving anyone at this stage [34].

#### Co-creation — Phase 4: Validation

The validation phase was performed in 5 of the 11 studies included in this systematic review [28,31,32,34,36]. Of the remaining studies, there was no validation phase in 2 studies [30,37]. In 2 studies [35,38], the final prototype was tested with a controlled trial, but the trial was not described. In 1 study, a basic evaluation of the prototype was done by the co-creation group, and a real-world study was planned but not performed [29]. In the other study [33], the protocol included a usability and user satisfaction study through a think-aloud method [40] and the IBM usability questionnaire [41,42] with 5 caregivers and 5 community workers, but this study was not completed.

A qualitative test of the prototype with a small sample of participants (2 or 3 patients and 2 caregivers) was included in 2 studies [31,36]. In 1 study [36], the experience and efficacy of the tool were analyzed, and in the other study [31], the authors used the Assessment of Autonomy in Internet-Mediated Activity tool [43] for activity performance analysis and breakdown detection, the Raw NASA Task Load Index questionnaire [44] to explore the task load of using the tool, and the System Usability Scale questionnaire [45] to analyze the user experience.

The other 3 studies performed a pilot test with a significant sample size [28,32,34]. In 1 study [32], the pilot was done in parallel in 3 sites with a sample size of 25 family caregivers and 25 community caregivers at each site, and the study explored application usage activity, acceptability, views on the app processes, and future refinements through qualitative data collection obtained in semistructured individual interviews. The last 2 studies performed a pilot to test feasibility and acceptability. One of the studies [32] was a pilot RCT with 20 participants (relatives and caregivers) in each arm examining the feasibility of recruiting and retaining participants; collecting relevant outcome data; and evaluating the intervention's acceptability, accessibility, and utility. Standardized questionnaires (Culturally adapted Knowledge About Psychosis questionnaire [46], Attitudes to Severe Mental Illness scale [47], SF-12 quality of life questionnaire [48]) were used for quantitative outcome measures, and semistructured individual interviews were used to collect qualitative acceptability data and to explore accessibility, usefulness, impact on attitudes and beliefs about schizophrenia, and feasibility aspects. The second study [28] was a single-arm feasibility pilot test with a sample of 30 patients, 18 caregivers, and 20 health professionals to test feasibility and acceptability and to refine the method for a larger study. Application usage was measured, baseline and follow-up surveys were used, semistructured interviews were conducted, and the results were summarized using a standardized index (Technology Readiness Index: A Multiple-Item Scale to Measure Readiness to Embrace New Technologies [49]) and content analysis.

Regarding agents involved in this phase, caregivers were involved, with the difference compared with the other agents, in 6 of the 11 articles [28,29,31-33,36], followed by health professionals [28,29,32,33] in 4 of the 11 articles; patients [28,31,36] in 3 of the 11 articles; and technology professionals [36], the research team [33], community members [33], social professionals [29], and museum managers [36] in at least 1 article each. In addition, 2 articles did not mention involving agents in this phase [30,37].

## Discussion

### Principal Findings

The objective of this systematic review was to identify co-created technological solutions, as well as the process or methodology followed for their co-creation, in the field of health for caregivers. The interventions in the included studies highlighted that, despite the rapid increase in interest in technological support for caregivers, very few studies included caregivers in the co-design process. In other reviews for specific populations such as older adults [50], none of the identified studies involved co-design or patient-oriented research approaches. The lack of standardized methodologies and the diversity of frameworks used in the co-creation process of technology solutions make it difficult to analyze and compare. Most of the technological solutions proposed in these studies were mobile or web applications, and all studies included caregivers, at least during design or development, and reached the prototyping phase of the technological solution.

### Technological Solutions

Regarding the type of technological solution designed and developed in the studies included in the review, in 9 of 11 articles, the proposal was a mobile or web application [28-30,32-37]. The proposals in the remaining 2 studies were based on high-tech products that involve more complex technologies such as augmented reality, robotics, or innovative assistive technologies (eg, sensor-based surveillance and monitoring systems, mobile technology such as wearable fall detectors) [31,35]. This could be due to the accessibility of mobile devices, mobile applications, and web applications and their ability to provide interventions instantly to promote health [51]. This allows and facilitates, in turn, intervening and interacting with users in their daily life and context, known as an ecological momentary intervention (EMI) [52-54]. EMI consists of momentary treatments provided via mobile technologies while people are engaged in their typical daily life routines [55]. EMI can be a useful add-on to traditional treatment, thanks to the 24-hour availability, low cost, and possibility of continuing follow-up in a nonpresential manner [56].

In turn, mobile or web applications have proven to be one of the most feasible technological solutions in digital health interventions with different population profiles [57-60]. In addition, previous studies suggest that mobile applications have the potential to have a greater positive impact on caregivers by providing support, communication, and facilitation of care, reducing the burden and positively impacting caregiver health outcomes [50].

On the other hand, the more significant presence of the design and development of mobile or web applications in this systematic review, compared with other types of technological solutions such as virtual reality, augmented reality, or robotics, could be due to the presence of this technology in people’s daily lives and, therefore, their knowledge, experience, and familiarity with it to actively participate in the process of co-creation [61].

Previous literature provides evidence that technology offers a cost-effective and practical method for delivering interventions to caregivers [62]. Nevertheless, the relevance of barriers to high-tech products suggests that external constraints impair consumers’ participation in complex or technologically advanced products [63]. Caregivers, possibly due to older age or little experience with these more complex technologies, do not realize how they can benefit personally from this kind of technology [64]. High-tech products require effort to obtain new specialized knowledge and skills. For this reason, we think that, in this case, it is even more important to involve caregivers from the beginning of the co-creation process in order to fulfill their needs and preferences. For example, robots have the potential to help with the caregiving and domestic needs of the growing aging population [65], and it is important to ensure the participation of caregivers in the creation of the solutions.

As some studies identified, almost one-half of the caregivers providing substantial help with health care assisted an older adult with dementia [66]. In this review, we identified that the needs of caregivers being tackled by technological solutions are very diverse. Even though there is research on the most common needs for caregivers of people with Alzheimer disease and dementia, as an example [67], their needs were related to personal health and receiving help from others, as well as information gaps and their education or learning needs. Although we can see how the articles identified in this study aimed to cover the needs related to information gaps and the education or learning needs [28-30,34,37,38], they also had other objectives that may not be a priority need for caregivers. Caregivers still have needs related to the action of caring, but none of the studies focused on one of the most important needs, such as caring for their health.

### Co-creation Process

This review also analyzed the co-creation process of solutions in which at least a functional prototype had been developed. Most of the existing published literature on this topic, including the recent [68], did not go beyond the design phase, which makes it difficult to understand the implementation and evaluation of co-creation processes and the effectiveness of the proposed solution.

The lack of standardized methodologies and diversity of frameworks used in the co-creation process of technology solutions make it difficult to analyze and classify the studies. The same occurred for other related, excluded studies, which used multimethod designs [69]. Also, the lack of information makes it impossible to replicate some of this work. In some studies, the project was developed following the waterfall model, which consists of linear-sequential phases that each depends on the completion of the previous phase. Currently, this model has also been used in other health care solutions focused on patients rather than caregivers [70,71]. However, in most of the studies, the execution of these phases is not sequential, and the development phase involves refining the previous phases. Moreover, 1 study followed an agile scrum co-design methodology [72] based on the prototype model; in this methodology, development occurs through short and fast iterations involving both co-design and development. Some authors conclude that the use of the scrum framework in health solutions efficiently helps to carry out activities by allowing careful analysis of each stage with regard to quality, technology, and implementation [73].

To conduct this review, we analyzed the studies through the 4 common phases of a development process: obtention of the requirements, design of the proposal, development of the prototype or product, and validation [27]. Regarding the requirements phase, some of the included studies detailed how the co-creation process was carried out. The main methods were interviews, workshops, expert meetings, surveys, and focus groups. The current literature indicates that qualitative studies using a focus group are a methodology widely used to engage stakeholders during co-creation [74]. Regarding the design phase, in 2 studies, a design phase was not detailed, but caregivers participated in workshops to co-create content and functionality in the development phase. The involvement of end users in the design process is known to be immensely valuable and facilitates a design process that is intuitive and attuned to the end users’ needs [75]. The development phase was detailed in most studies, and caregivers (and other participants) were involved. The involvement of co-creation agents varied from testing feedback sessions to participating in workshops to create and select content and functionality. In the studies in which validation was detailed, a pilot with a significant sample of participants and a validation of the prototype with a small sample were conducted. Few of these studies use standardized questionnaires. The case study methodology is another tool found in the literature to understand which co-created solutions work effectively [76].

In a co-creation process in which caregivers are the target population, it would be expected that they would be involved in all 4 phases of the process. However, of the 11 studies analyzed, in only 2 cases [33,35], caregivers were involved in all phases, although the studies did not describe the validation phase or its results but simply stated that a usability test or a pilot was carried out at a later stage. Nevertheless, the current literature indicates that end users should be extensively involved, in many different roles, which will give them the opportunity to not only evaluate each phase but also enlarge their involvement in the eventual product [75].

Regarding the analysis of the effectiveness of the use of co-created technological solutions by caregivers, with this systematic review, we could not determine the long-term outcomes of the identified projects. Most of the articles were published less than 2 years before the search for this study was carried out, so this could be the main reason why experimental studies were not found to analyze the impact of the use of the proposed technological solution. In addition, 2 of the included studies were published in 2011 [30,35]. In these cases, it is possible that experimental studies used co-created technology solutions, but they were not returned in the search because the co-creation process was not the focus of the study. The study by Lemetyinen et al [34] is the only experimental study that met the requirements, but they did not explain their co-creation process, and the previous study that detailed the process was not found in our search. The web application on which the intervention was based is not currently operational. Considering the timeliness of the studies included in this systematic review, it could be of great interest in the future to carry out a study that allows determining the success rate of the development and implementation of the technology, the analysis of its effectiveness, and if the technology was offered to the general public. Since this is the purpose of the design and development of technological solutions, it is well known that numerous factors can intercede in this process, starting with economic factors.

After analyzing the 11 studies, the future of technology solutions for caregivers need to focus on (1) identifying the common needs of caregivers, regardless of for whom they care, to be able to create specific solutions with them; (2) providing more detailed information on the creation process, because if caregivers were involved, this can add value for its use; (3) involve caregivers more actively at all stages of the creative process, as this can substantially increase the usefulness of the created technologies; and (4) use validated tools regularly and to increase the scientific evidence on the impact of the technological solutions created.

### Strengths and Limitations

The main strengths of this review are based on the multiple steps performed to achieve methodological rigor. The review was guided by PRISMA, and the database searches, screening, data extraction, methodological assessment, and level of evidence evaluations were conducted in duplicate, with strong agreement between reviewers. In addition, the search was conducted in 2 languages, English and Spanish, which allowed for a broader review of the literature. Finally, as far as we know, this is the first review focused on co-created technological solutions for caregivers in health care.

Regarding the limitations, although appropriate keywords were used, there may be a certain word from a specific area that has not been checked. Consultation with a librarian could have helped. Another limitation of the systematic review is that a meta-analysis could not be performed since heterogeneous studies with poor methodological quality and limited results emerged. Future research using validated tools is needed to evaluate the technological solutions for a more in-depth analysis.

### Conclusions

In summary, the current systematic review suggests that, despite the increasing need to provide technological support for informal caregivers, very few studies included them in the co-creation process. The existing co-created technological solutions in the health field for caregivers are mostly mobile or web applications aimed at supporting caregivers' social health and well-being and improve their health knowledge when delivering care to patients, most commonly older people. As for the co-creation process, caregivers are more likely to be involved at the time of development and in the design. Future research should include the following criteria: detailed reporting on the co-creation process, involving caregivers more actively in all phases of the process, and using validated tools to evaluate the impact of the technological solutions created. Scientific evidence could help informal caregivers in their caregiving tasks.

## Appendix

Multimedia Appendix 1 Search terms.

Multimedia Appendix 2 Methodological quality assessment of the included articles.

Multimedia Appendix 3 Detailed phases of the co-creation process in the included articles.

## Abbreviations

EMI: ecological momentary intervention
PRSIMA: Preferred Reporting Items for Systematic Reviews and Meta-Analysis
RCT: randomized controlled trial
SIGN: Scottish Intercollegiate Guidelines Network

## References

[1] Adelman RD, Tmanova LL, Delgado D, Dion S, Lachs MS (2014). Caregiver burden: a clinical review. JAMA.

[2] (2022). Healthcare expenditure, UK Health Accounts: 2020. Office for National Statistics.

[3] Zigante V, Directorate-General for Employment, Social Affairs and Inclusion (European Commission), London School of Economics and Political Science (LSE) (2018). Informal care in Europe: exploring formalisation, availability and quality. Publications Office of the European Union.

[4] Disability and Carers. Australian Government Department of Social Services.

[5] Besnard X, Brunel M, Couvert N, Roy D (2015). Caregivers of seniors and their feelings about the help provided - Results of the "CARE" surveys of caregivers (2015-2016). Directorate of Research, Studies, Evaluation and Statistics.

[6] (2020). Caregiving in the U.S. 2020: A Focused Look at Family Caregivers of Adults Age 18 to 49. The National Alliance for Caregiving.

[7] Lopez-Hartmann M, Wens J, Verhoeven V, Remmen R (2012). The effect of caregiver support interventions for informal caregivers of community-dwelling frail elderly: a systematic review. Int J Integr Care.

[8] Brown EL, Ruggiano N, Page TF, Roberts L, Hristidis V, Whiteman KL, Castro J (2016). CareHeroes web and Android™ apps for dementia caregivers: a feasibility study. Res Gerontol Nurs.

[9] Vaingankar JA, Chong SA, Abdin E, Picco L, Jeyagurunathan A, Zhang Y, Sambasivam R, Chua BY, Ng LL, Prince M, Subramaniam M (2016). Care participation and burden among informal caregivers of older adults with care needs and associations with dementia. Int Psychogeriatr.

[10] Aksoydan E, Aytar A, Blazeviciene A, van Bruchem-Visser RL, Vaskelyte A, Mattace-Raso F, Acar S, Altintas A, Akgun-Citak E, Attepe-Ozden S, Baskici C, Kav S, Kiziltan G (2019). Is training for informal caregivers and their older persons helpful? A systematic review. Arch Gerontol Geriatr.

[11] Mortenson WB, Demers L, Fuhrer MJ, Jutai JW, Lenker J, DeRuyter F (2012). How assistive technology use by individuals with disabilities impacts their caregivers: a systematic review of the research evidence. Am J Phys Med Rehabil.

[12] Broderick J, Devine T, Lemerise Aj, Lier S, Harris L (2014). Designing health literate mobile apps. NAM Perspectives.

[13] Hochstenbach LMJ, Courtens AM, Zwakhalen SMG, Vermeulen J, van Kleef M, de Witte LP (2017). Co-creative development of an eHealth nursing intervention: Self-management support for outpatients with cancer pain. Appl Nurs Res.

[14] Kalem G, Turhan (2015). Mobile technology applications in the healthcare industry for disease management and wellness. Procedia - Social and Behavioral Sciences.

[15] Ward ME, De Brún A, Beirne D, Conway C, Cunningham U, English A, Fitzsimons J, Furlong E, Kane Y, Kelly A, McDonnell S, McGinley S, Monaghan B, Myler A, Nolan E, O'Donovan R, O'Shea M, Shuhaiber A, McAuliffe E (2018). Using co-design to develop a collective leadership intervention for healthcare teams to improve safety culture. Int J Environ Res Public Health.

[16] Bate P, Robert G (2006). Experience-based design: from redesigning the system around the patient to co-designing services with the patient. Qual Saf Health Care.

[17] Bovaird T, Loeffler E (2012). From engagement to co-production: the contribution of users and communities to outcomes and public value. Voluntas.

[18] Cho HJ, Smith D, Hart A, Prasad R, Sata SS, Clarke K, Arole O, Beurlein J, George M, Moore C, Schleyer AM, Wooldridge K, Wosk TB, Yousef E, Goldstein J, Fegley AE, Malouk M, Krouss M (2022). Choosing wisely in adult hospital medicine: co-creation of new recommendations for improved healthcare value by clinicians and patient advocates. J Gen Intern Med.

[19] Ghosh S, Struminger BB, Singla N, Roth BM, Kumar A, Anand S, Mtete E, Lusekelo J, Massawe I, Jarpe-Ratner E, Seweryn SM, Risley K, Moonan PK, Pinsker E (2022). Appreciative inquiry and the co-creation of an evaluation framework for Extension for Community Healthcare Outcomes (ECHO) implementation: a two-country experience. Eval Program Plann.

[20] Langley J, Wolstenholme D, Cooke J (2018). 'Collective making' as knowledge mobilisation: the contribution of participatory design in the co-creation of knowledge in healthcare. BMC Health Serv Res.

[21] Bruvik FK, Ulstein ID, Ranhoff AH, Engedal K (2012). The quality of life of people with dementia and their family carers. Dement Geriatr Cogn Disord.

[22] Zacharopoulou G, Zacharopoulou V, Lazakidou A (2015). Quality of life for caregivers of elderly patients with dementia and measurement tools: a review. International Journal Of Health Research and Innovation.

[23] Boyd H, McKernon S, Mullin B, Old A (2012). Improving healthcare through the use of co-design. N Z Med J.

[24] Dickson K, Yeung CA (2022). PRISMA 2020 updated guideline. Br Dent J.

[25] Harbour R, Miller J (2001). A new system for grading recommendations in evidence based guidelines. BMJ.

[26] Pace R, Pluye P, Bartlett G, Macaulay AC, Salsberg J, Jagosh J, Seller R (2012). Testing the reliability and efficiency of the pilot Mixed Methods Appraisal Tool (MMAT) for systematic mixed studies review. Int J Nurs Stud.

[27] Leask CF, Sandlund M, Skelton DA, Altenburg TM, Cardon G, Chinapaw MJM, De Bourdeaudhuij I, Verloigne M, Chastin SFM, GrandStand, Safe Step and Teenage Girls on the Move Research Groups (2019). Framework, principles and recommendations for utilising participatory methodologies in the co-creation and evaluation of public health interventions. Res Involv Engagem.

[28] Backman C, Harley A, Kuziemsky C, Mercer J, Peyton L (2020). MyPath to Home web-based application for the geriatric rehabilitation program at Bruyère Continuing Care: user-centered design and feasibility testing study. JMIR Form Res.

[29] Egan K, Hodgson W, Dunlop M, Imperatore G, Kirk A, Maguire R (2021). A novel mobile app ("CareFit") to support informal caregivers to undertake regular physical activity from home during and beyond COVID-19 restrictions: co-design and prototype development study. JMIR Form Res.

[30] de la Harpe R, van Zyl I (2011). Community 2.0: A collaborative university-community network to inform and educate caregivers in home-based healthcare in South Africa.

[31] Guerrero E, Lu M, Yueh H, Lindgren H (2019). Designing and evaluating an intelligent augmented reality system for assisting older adults’ medication management. Cognitive Systems Research.

[32] Harding R, Carrasco JM, Serrano-Pons J, Lemaire J, Namisango E, Luyirika E, Immanuel T, Paleri AK, Mathews L, Chifamba D, Mupaza L, Martínez CL, Zirimenya L, Bouësseau MC, Krakauer EL (2021). Design and evaluation of a novel mobile phone application to improve palliative home-care in resource-limited settings. J Pain Symptom Manage.

[33] Latulippe K, Hamel C, Giroux D (2020). Integration of conversion factors for the development of an inclusive eHealth tool with caregivers of functionally dependent older persons: social justice design. JMIR Hum Factors.

[34] Lemetyinen H, Onwumere J, Drake R, Abel K, Haigh C, Moulton G, Edge D (2018). Co-production and evaluation of an e-learning resource to improve African-Caribbean families' knowledge about schizophrenia and engagement with services: a pilot randomised controlled trial protocol. Pilot Feasibility Stud.

[35] Meiland FJM, Hattink BJJ, Overmars-Marx T, de Boer ME, Jedlitschka A, Ebben PWG, Stalpers-Croeze IINW, Flick S, van der Leeuw J, Karkowski IP, Dröes RM (2014). Participation of end users in the design of assistive technology for people with mild to severe cognitive problems; the European Rosetta project. Int Psychogeriatr.

[36] O'Connor S (2020). Co-designing technology with people with dementia and their carers: Exploring user perspectives when co-creating a mobile health application. Int J Older People Nurs.

[37] Rathnayake S, Moyle W, Jones C, Calleja P (2021). Co-design of an mHealth application for family caregivers of people with dementia to address functional disability care needs. Inform Health Soc Care.

[38] Sin J, Henderson C, Woodham LA, Sesé Hernández A, Gillard S (2019). A multicomponent eHealth intervention for family carers for people affected by psychosis: a coproduced design and build study. J Med Internet Res.

[39] Vargo SL, Lusch RF (2018). Evolving to a new dominant logic for marketing. Journal of Marketing.

[40] Jaspers MWM, Steen T, van den Bos C, Geenen M (2004). The think aloud method: a guide to user interface design. Int J Med Inform.

[41] Lewis JR (1995). IBM computer usability satisfaction questionnaires: Psychometric evaluation and instructions for use. International Journal of Human-Computer Interaction.

[42] Yen P, Bakken S (2012). Review of health information technology usability study methodologies. J Am Med Inform Assoc.

[43] Lindgren H (2009). Personalisation of internet-mediated activity support systems in the rehabilitation of older adults: a pilot study. AIME 2009 International Workshop on Personalisation for e-Health.

[44] Grier RA (2016). How High is High? A Meta-Analysis of NASA-TLX Global Workload Scores. Proceedings of the Human Factors and Ergonomics Society Annual Meeting.

[45] Brooke J, Jordan PW, Thomas B, McClelland IL, Weerdmeester B (1996). SUS - a quick and dirty usability scale. Usability Evaluation In Industry.

[46] Degnan A, Berry K, James S, Edge D (2018). Development, validation and cultural-adaptation of the knowledge about psychosis questionnaire for African-Caribbean people in the UK. Psychiatry Res.

[47] Madianos M, Economou M, Peppou LE, Kallergis G, Rogakou E, Alevizopoulos G (2012). Measuring public attitudes to severe mental illness in Greece: Development of a new scale. Eur. J. Psychiat.

[48] Ware J, Kosinski M, Keller SD (1996). A 12-Item Short-Form Health Survey: construction of scales and preliminary tests of reliability and validity. Med Care.

[49] Parasuraman A, Colby CL (2014). An updated and streamlined technology readiness index. Journal of Service Research.

[50] Garnett A, Northwood M, Ting J, Sangrar R (2022). mHealth interventions to support caregivers of older adults: equity-focused systematic review. JMIR Aging.

[51] McKay FH, Wright A, Shill J, Stephens H, Uccellini M (2019). Using health and well-being apps for behavior change: a systematic search and rating of apps. JMIR Mhealth Uhealth.

[52] Hawker CO, Merkouris SS, Youssef GJ, Dowling NA (2021). A smartphone-delivered ecological momentary intervention for problem gambling (GamblingLess: Curb Your Urge): single-arm acceptability and feasibility trial. J Med Internet Res.

[53] Hanssen E, Balvert S, Oorschot M, Borkelmans K, van Os J, Delespaul P, Fett A (2020). An ecological momentary intervention incorporating personalised feedback to improve symptoms and social functioning in schizophrenia spectrum disorders. Psychiatry Res.

[54] Smith KE, Juarascio A (2019). From ecological momentary assessment (EMA) to ecological momentary intervention (EMI): past and future directions for ambulatory assessment and interventions in eating disorders. Curr Psychiatry Rep.

[55] Dias N, Boring E, Johnson LA, Grossoehme DH, Murphy S, Friebert S (2021). Developing a theoretically grounded, digital, ecological momentary intervention for parental bereavement care using the ORBIT model-Phase 1. Death Stud.

[56] Barrigon ML, Porras-Segovia A, Courtet P, Lopez-Castroman J, Berrouiguet S, Pérez-Rodríguez MM, Artes A, Baca-Garcia E, MEmind Study Group (2022). Smartphone-based Ecological Momentary Intervention for secondary prevention of suicidal thoughts and behaviour: protocol for the SmartCrisis V.2.0 randomised clinical trial. BMJ Open.

[57] Nesvåg S, McKay JR (2018). Feasibility and effects of digital interventions to support people in recovery from substance use disorders: systematic review. J Med Internet Res.

[58] Saad A, Bruno D, Camara B, D'Agostino J, Bolea-Alamanac B (2021). Self-directed technology-based therapeutic methods for adult patients receiving mental health services: systematic review. JMIR Ment Health.

[59] Stokes K, Oronti B, Cappuccio FP, Pecchia L (2022). Use of technology to prevent, detect, manage and control hypertension in sub-Saharan Africa: a systematic review. BMJ Open.

[60] Berkanish P, Pan S, Viola A, Rademaker Q, Devine KA (2022). Technology-based peer support interventions for adolescents with chronic illness: a systematic review. J Clin Psychol Med Settings.

[61] Lavallee DC, Lee JR, Semple JL, Lober WB, Evans HL (2019). Engaging patients in co-design of mobile health tools for surgical site infection surveillance: implications for research and implementation. Surg Infect (Larchmt).

[62] Finkel S, Czaja SJ, Schulz R, Martinovich Z, Harris C, Pezzuto D (2007). E-care: a telecommunications technology intervention for family caregivers of dementia patients. Am J Geriatr Psychiatry.

[63] Mandolfo M, Chen S, Noci G (2020). Co-creation in new product development: Which drivers of consumer participation?. International Journal of Engineering Business Management.

[64] Thordardottir B, Malmgren Fänge A, Lethin C, Rodriguez Gatta D, Chiatti C (2019). Acceptance and use of innovative assistive technologies among people with cognitive impairment and their caregivers: a systematic review. Biomed Res Int.

[65] Hall AK, Backonja U, Painter I, Cakmak M, Sung M, Lau T, Thompson HJ, Demiris G (2019). Acceptance and perceived usefulness of robots to assist with activities of daily living and healthcare tasks. Assist Technol.

[66] Wolff JL, Spillman BC, Freedman VA, Kasper JD (2016). A national profile of family and unpaid caregivers who assist older adults with health care activities. JAMA Intern Med.

[67] Queluz FNFR, Kervin E, Wozney L, Fancey P, McGrath PJ, Keefe J (2020). Understanding the needs of caregivers of persons with dementia: a scoping review. Int Psychogeriatr.

[68] Tiersen F, Batey P, Harrison MJC, Naar L, Serban A, Daniels SJC, Calvo RA (2021). Smart home sensing and monitoring in households with dementia: user-centered design approach. JMIR Aging.

[69] Strudwick G, McLay D, Lo B, Shin HD, Currie L, Thomson N, Maillet ?, Strong V, Miller A, Shen N, Campbell J (2021). Development of a resource guide to support the engagement of mental health providers and patients with digital health tools: multimethod study. J Med Internet Res.

[70] Abba S, Wadumi Namkusong J, Lee JA, Liz Crespo M (2019). Design and performance evaluation of a low-cost autonomous sensor interface for a smart IoT-based irrigation monitoring and control system. Sensors (Basel).

[71] Pedraza LL, Moraes JRWD, Rabelo-Silva ER (2020). Development and testing of a text messaging (SMS) monitoring software application for acute decompensated heart failure patients. Rev Lat Am Enfermagem.

[72] Klein L Agile Development. Interaction Design Foundation.

[73] Torrente G, de Souza TQ, Tonaki L, Cardoso AP, Manickchand Junior L, da Silva GO (2021). Scrum framework and health solutions: management and results. Stud Health Technol Inform.

[74] Ariza-Vega P, Prieto-Moreno R, Mora-Traverso M, Molina-García P, Ashe MC, Martín-Matillas M (2022). Co-creation of mHealth intervention for older adults with hip fracture and family caregivers: a qualitative study. Disabil Rehabil Assist Technol.

[75] Korving H, Sterkenburg PS, Barakova EI, Feijs LMG (2022). Designing pain visualisation for caregivers of people with special needs: A co-creation approach. Heliyon.

[76] Smith G, Dixon C, Neiva Ganga R, Greenop D (2022). How do we know co-created solutions work effectively within the real world of people living with dementia? Learning methodological lessons from a co-creation-to-Evaluation Case Study. Int J Environ Res Public Health.

