# Supplementary material for: Co-created Technological Solutions for Caregivers in Health Care: Systematic Review
Source: J Med Internet Res. 2023 May 1;25:e41260. doi: 10.2196/41260 (PMC10186187; doi:10.2196/41260)
Supplement: Multimedia Appendix 1 [file jmir_v25i1e41260_app1.docx]

**Multimedia Appendix 1: Search terms**

(co-creation OR co-design OR co-production OR “participatory design” OR user-led) AND (digital OR “web application” OR “web app” OR virtual OR online OR technology OR “artificial intelligence” OR “machine learning” OR blockchain OR cloud OR cybersecurity OR hardware OR “internet of things” OR robotics OR “3D printing” OR “big data” OR games OR mobile OR drones OR “augmented reality” OR “mixed reality”) AND (health OR healthcare OR e-health OR ehealth OR m-health OR mhealth OR healthtech OR telehealth) AND (carer OR caregiver).
